# Skeletal Muscle Microvascular Changes in Response to Short-Term Blood Flow Restricted Training—Exercise-Induced Adaptations and Signs of Perivascular Stress

**DOI:** 10.3389/fphys.2020.00556

**Published:** 2020-06-12

**Authors:** Jakob L. Nielsen, Ulrik Frandsen, Kasper Y. Jensen, Tatyana A. Prokhorova, Line B. Dalgaard, Rune D. Bech, Tobias Nygaard, Charlotte Suetta, Per Aagaard

**Affiliations:** ^1^Department of Sports Science and Clinical Biomechanics and SDU Muscle Research Cluster, Faculty of Health Sciences, University of Southern Denmark, Odense, Denmark; ^2^Section for Sports Science, Department of Public Health, Faculty of Health, Aarhus University, Aarhus, Denmark; ^3^Department of Orthopaedic Surgery, Rigshospitalet, University of Copenhagen, Copenhagen, Denmark; ^4^Geriatric Research Unit, Department of Geriatric and Palliative Medicine, Bispebjerg-Frederiksberg Hospital, University of Copenhagen, Copenhagen, Denmark; ^5^Geriatric Research Unit, Department of Medicine, Herlev-Gentofte Hospital, University of Copenhagen, Copenhagen, Denmark

**Keywords:** angiogenesis, capillary, hypoxia, vascular remodeling, vascular stress

## Abstract

**Aim:** Previous reports suggest that low-load muscle exercise performed under blood flow restriction (BFR) may lead to endurance adaptations. However, only few and conflicting results exist on the magnitude and timing of microvascular adaptations, overall indicating a lack of angiogenesis with BFR training. The present study, therefore, aimed to examine the effect of short-term high-frequency BFR training on human skeletal muscle vascularization.

**Methods:** Participants completed 3 weeks of high-frequency (one to two daily sessions) training consisting of either BFR exercise [(BFRE) *n* = 10, 22.8 ± 2.3 years; 20% one-repetition maximum (1RM), 100 mmHg] performed to concentric failure or work-matched free-flow exercise [(CON) *n* = 8, 21.9 ± 3.0 years; 20% 1RM]. Muscle biopsies [vastus lateralis (VL)] were obtained at baseline, 8 days into the intervention, and 3 and 10 days after cessation of the intervention to examine capillary and perivascular adaptations, as well as angiogenesis-related protein signaling and gene expression.

**Results:** Capillary per myofiber and capillary area (CA) increased 21–24 and 25–34%, respectively, in response to BFRE (*P* < 0.05–0.01), while capillary density (CD) remained unchanged. Overall, these adaptations led to a consistent elevation (15–16%) in the capillary-to-muscle area ratio following BFRE (*P* < 0.05–0.01). In addition, evaluation of perivascular properties indicated thickening of the perivascular basal membrane following BFRE. No or only minor changes were observed in CON.

**Conclusion:** This study is the first to show that short-term high-frequency, low-load BFRE can lead to microvascular adaptations (i.e., capillary neoformation and changes in morphology), which may contribute to the endurance effects previously documented with BFR training. The observation of perivascular membrane thickening suggests that high-frequency BFRE may be associated with significant vascular stress.

## Introduction

During the last decades, BFR exercise (BFRE) has been established as a low-load training modality able of stimulating gains in skeletal muscle mechanical contractile properties (e.g., maximal and explosive muscle strength) and skeletal muscle mass (Takarada et al., 2000; Kacin and Strazar, 2011; Nielsen et al., 2012, 2017b). In addition to gains in maximal muscle strength, studies have reported improved endurance capacity; i.e., increased number of repetitions to fatigue in young recreational active individuals after 4-week BFR resistance training (Kacin and Strazar, 2011), as well as improved VO_2_ max (Abe et al., 2010) and power output during incremental all-out knee extensor testing (Christiansen et al., 2019) in untrained individuals following 6–8 weeks of BFR cycling. Such endurance effects have been suggested to partly be attributed to increased O_2_ delivery to the trained musculature after BFR training (Kacin and Strazar, 2011). The potential of BFR training to evoke adaptative gains in O_2_ delivery and/or local blood flow is indirectly supported by reports of increased muscle filtration capacity after short-term (4 weeks) BFR training (Evans et al., 2010; Hunt et al., 2013), given that changes in tissue filtration capacity can reflect improvements in microvascular capillarization (Brown et al., 2001).

*In vivo* skeletal muscle angiogenesis is controlled by a complex signaling network in which vascular endothelial growth factor (VEGF) is considered a key regulator of capillary splitting and sprouting (Wagner, 2011; Hoier and Hellsten, 2014). Skeletal muscle tissue angiogenesis as well as VEGF secretion and release are thought to be related to cellular hypoxia, increased myocellular metabolism, vascular endothelial shear stress, and/or dynamic stretch of the vascular wall (Egginton, 2009). Notably, all of these factors may be induced by BFRE, as evidence of local tissue hypoxia, accumulation of metabolites, venous blood pooling, and substantial reperfusion have been reported with this training modality (Takano et al., 2005; Larkin et al., 2012; Takada et al., 2012). Recently, elevated mRNA transcripts of several angiogenesis-related genes were demonstrated 4–24 h after acute BFRE, with VEGF demonstrating a particular profound increase (four- to sixfold) (Larkin et al., 2012; Ferguson et al., 2018). Conversely, a lack of parallel increases in VEGF protein content in muscle or serum has also been reported (Larkin et al., 2012). Nevertheless, increases in plasma VEGF have been reported in young and old male individuals in the early phase (0–120 min) after acute BFRE (Takano et al., 2005; Patterson et al., 2013). Taken together, these data support a potential stimulation of angiogenesis with longitudinal BFR training. Surprisingly, 6 weeks of body weight BFR training or 4 weeks of sprint interval training followed by post-exercise passive BFR did not appear to stimulate angiogenesis, as determined by capillary per fiber (C:F) and capillary density (CD) (Jakobsgaard et al., 2018; Mitchell et al., 2019). Illustrating the conflicting observations of vascular plasticity with BFR training, an increased C:F ratio in type I myofibers recently was reported following 6 weeks of alternating low-load BFR training and high-load free-flow training in highly trained elite powerlifters (Bjornsen et al., 2019).

Consequently, the aim of the present study was to investigate the effect of short-term low-load BFR resistance training on vascular properties and angiogenic signaling in human skeletal muscle. To investigate myocellular factors potentially responsible for initiating the angiogenic response, secondary analyses of muscle protein and gene expression biomarkers known to stimulate angiogenesis and extracellular matrix (ECM) remodeling were also performed. As an indirect marker of accumulated vascular stress, perivascular ECM morphology was qualitatively examined.

We hypothesized that BFR training would lead to an amplified angiogenic response compared to free-flow work-matched exercise conditions.

## Materials and Methods

Twenty-one healthy male participants volunteered to participate in the study. Participants were divided into a BFRE training group (*n* = 12; age 22.8 ± 2.1 years; height 181.2 ± 6.4 cm; body mass 82.3 ± 13.7 kg) and a control group (CON) (*n* = 9; age 21.9 ± 3.0 years; height 182.9 ± 8.8 cm; body mass 80.2 ± 11.4 kg). A number of participants (BFRE = 2/CON = 1) left the study prematurely (see more details in section “Participants”), leaving 10 and eight participants in the BFRE and CON groups, respectively. All participants were recreationally active and had not participated in any systematic strength training within a year prior to the study. The study was approved by the local Ethics Committee (Region of Southern Denmark) (S-200900070) in accordance with the Helsinki Declaration, and written informed consent was obtained from all participants prior to inclusion. Data from this study have been published previously (Nielsen et al., 2012, 2017a,b).

### Study Protocol

Muscle biopsy samples were obtained from vastus lateralis (VL) muscle before (Pre) and 8 days (Mid8) into the intervention as well as 3 and 10 days after cessation of training (Post3 and Post10, respectively). Biopsy samplings were performed in the experimental (EX) leg, which was chosen by paired randomization between legs. At least 1 week prior to testing, participants visited the laboratory for determination of one-repetition maximum (1RM). Participants were carefully instructed to maintain their normal daily routines in relation to nutritional intake and physical activity and refrain from alcohol intake during the intervention period. Furthermore, participants were asked not to participate in moderate to hard physical activity within 48 h prior to sampling procedures, which were conducted at the same time of the day to avoid diurnal variations.

### Training Protocol

The training protocol has been described in detail elsewhere (Nielsen et al., 2012). In brief, participants completed 23 separate supervised training sessions in 19 days (one to two daily sessions with weekends off). Each training session consisted of four sets of 20% of 1RM unilateral knee extensor exercise with 30-s pauses between sets. In each set, the BFRE participants performed repetitions to concentric failure with concurrent BFR, whereas CON performed a relative work-matched exercise protocol without BFR (i.e., in free-flow conditions). The CON exercise protocol was matched *a posteriori* to the exercise data (i.e., completed repetitions) of the BFRE participants. Verbal encouragement was applied in all training sessions to optimize voluntary effort. Partial BFR was applied in BFR using a pneumatic cuff (13.5 cm cuff width) (Delfi Medical, Canada) that was connected to a computerized system (Zimmer A.T.S. 750, United States) enabling automatic instantaneous regulation of occlusion pressure. Prior to each BFRE session, the cuff was placed around the proximal thigh (Nielsen et al., 2012) and inflated to 100 mmHg ∼10 s before the first set until completion of the fourth exercise set.

### Muscle Tissue Sampling and Handling

Muscle biopsy samples were obtained from muscle VL before training (Pre), 8 days into the intervention (Mid8), and 3 and 10 days after the intervention (Post3 and Post10, respectively) (Nielsen et al., 2012). Biopsy sampling was performed during local anesthesia (1% lidocaine, Amgros, Denmark) with a Bergström biopsy needle with suction. All biopsies were taken from the same muscle depth and in a randomized fashion approximately 3 cm apart. Pieces of muscle samples were mounted in Tissue-Tec (Sakura Finetek, Netherlands) and frozen in isopentane precooled in liquid nitrogen or frozen directly in liquid nitrogen. After the initial handling, all samples were stored at −80°C for future analysis. Protein array and mRNA expression analysis were only performed on BFRE samples, as there was insufficient muscle homogenate available for controls to ensure a high enough sample size.

### Immunofluorescence Analysis

In brief, transverse cut serial sections (8 μm) were fixed (4% formaldehyde, Triton-X buffer) and subsequently blocked (X0909, Dako, Glostrup, Denmark) for 10 min. Sections were incubated for 60 min in room temperature with primary antibodies: CD31 (1:100, M0823, Dako) and neural/glial antigen 2 (NG2, 1:200, B5320, Merck Millipore, Darmstadt, Germany), while subsequently incubated with secondary antibodies: DyLight-488 anti-mouse (Vector, DK-2488, Burlingame, CA, United States) and biotinylated anti-rabbit (1:200; Vector) followed by DyLight-549 Streptavidin (1:500, Vector). A second analysis was performed to visualize the basal lamina using laminin (Z0097, Dako; 1:1,000) and Alexa-488 goat-anti-mouse (A11029, TS; 1:1,000). Finally, sections were washed in PBS, mounted in medium containing 4’,6-diamidino-2-phenylindole (DAPI, Molecular Probes, P36935) and stored protected from light at 5°C. Sections in which primary antibodies were omitted served as negative controls.

**FIGURE 1:**
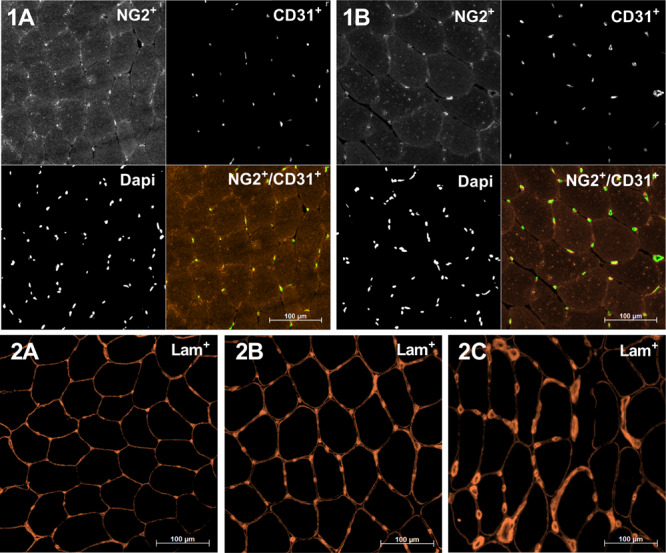
Representative skeletal muscle cross sections displaying immunoreactivity for NG2 (red), CD31 (green), and 4’,6-diamidino-2-phenylindole (DAPI) (nuclear stain) **(1A,1B)** and Laminin **(2A–2C)**. **1A,1B**: Samples are from blood flow restriction exercise (BFRE) at baseline (Pre, 1A) and 8 days into the intervention (Mid8, **1B**). Note the increase in the area of CD31C structures and myofibers as well as a similar number of CD31C structures, suggesting a stable capillary density (CD) and an increase in capillary/fiber and capillary cross-sectional area. **2A**: Normal laminin morphology, baseline (Pre); **2B/2C**: lowly/highly increased perivascular laminin immunoreactivity relative to baseline **(2A)**.

Stained sections were visualized using fluorescent microscopy (Axio Imager M1, Carl Zeiss, Germany) and a high-resolution AxioCam (Carl Zeiss). Capillaries and pericytes defined as CD31^+^ (green) and NG2^+^ (red) structures, respectively, were identified. The number of capillaries was expressed per 100 myofibers (C:F) and the CD was calculated as the number of capillaries per mm^2^. During previous myocellular analyses of samples from the same study (Nielsen et al., 2012, 2017a), we observed some distinct changes in perivascular basal lamina morphology. Consequently, an analysis evaluating perivascular basal lamina immunoreactivity (pBLi) was performed. A participant-specific baseline pBLi (Figure 1, 2A) was established, and focal changes in pBLi relative to baseline expression were defined as low, moderate, or high (Figure 1, 2B). These analyses were performed qualitatively, as the observed changes in perivascular basal lamina morphology were mostly focally presented, thus a quantitative analysis would not reveal these changes. The qualitative analysis was performed by two separate investigators (JN and UF). All these analyses were performed manually in AxioVision image analysis software (AxioVision 4.6, Carl Zeiss). In addition, the capillary area (CA; i.e., area of CD31 immunoreactivity) was analyzed in ImageJ 1.8 [National Institutes of Health (NIH), Bethesda, MD, United States] using the built-in threshold function. Data for any non-capillary structures (large vessels, auto-fluorescence, etc.) were carefully excluded using visual inspection. The mean CA and total CA to skeletal muscle area ratio (CMAR) were calculated. CD31^+^/DAPI^+^ (endothelial) and positive NG2^+^/DAPI^+^ cells (pericytes) were counted and normalized per myofiber and myofiber area. As it was not possible to distinguish endothelial cells and pericytes due to the close spatial location, these cells were most often evaluated together (e.g., CD31^+^/NG2^+^ cells). Then, 107 ± 22 myofibers (C:F, CD, NG2^+^), 637 ± 206 capillaries (CA, CAMR), and sections containing >120 (range: 122–232) myofibers (pBLi perivascular immunoreactivity) were analyzed per muscle biopsy. All analyses were performed by an investigator blinded to participant ID, group, and time of sampling. One BFR participant failed to meet up to the Mid8 biopsy sampling, making only nine data points available for this time point, while a dataset from one participant was omitted from CON due to inadequate biopsy histology.

**FIGURE 2:**
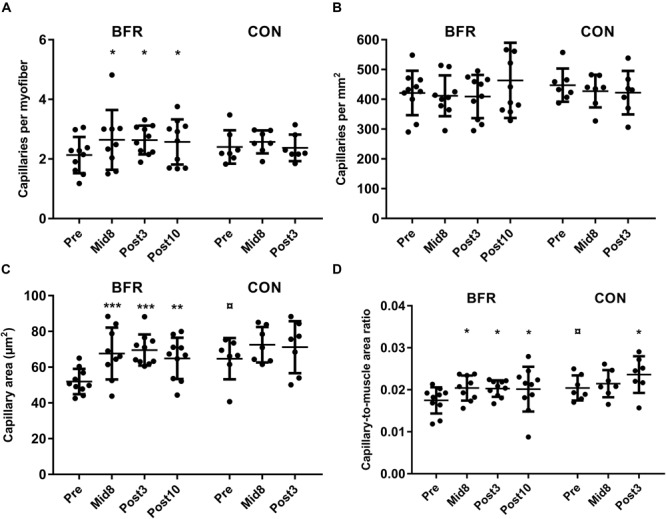
Capillaries per myofiber, capillary density (CD, capillaries per mm^2^), capillary cross-sectional area (μm^2^), and capillary-to-muscle area ratio at baseline (Pre), 8 days into the training intervention (Mid8), and 3 and 10 days after cessation of training (Post3 and Post10, respectively). **(A)** Capillaries per myofiber, **(B)** CD (capillaries per mm^2^), **(C)** average capillary area, and **(D)** capillary-to-muscle area ratio. Pre to Mid/post differences: *P < 0.05, **P < 0.01, ***P < 0.001. Baseline-specific group difference: *P <* 0.05. Values are means ± SD; BFR: *n* = 10 at Mid8 *n* = 9; CON: *n* = 7.

### Protein Array Analysis

Muscle samples were placed in ice-cold homogenizing buffer containing 200 mM sucrose, 1 mM ethylenediaminetetraacetic acid (EDTA), 10 mM sodium azide, 40 mM Tris-base (pH 7.8), protease inhibitor (5892791001, Sigma–Aldrich), after which it was gently homogenized in a glass homogenizer with a Teflon pestle. Samples were kept on ice during the entire procedure. Homogenates were analyzed for angiogenesis protein expression by human protein array analysis (Q1000; RayBiotech, Peachtree Corners, GA, United States) as per the manufacturer’s instructions.

### RNA Extraction and cDNA Synthesis

Tissue was homogenized in tubes containing 10 ceramic beads and one silicium crystal using the MagnaLyzer (Roche, Hvidovre, Denmark). Total RNA was extracted from homogenate using TRIzol Reagent (Life Technologies) according to manufacturer instructions. RNA concentration (ng/ml) and purity (260/280) were determined with the Nano-Drop Spectrophotometer (ND1000; Thermo Scientific, Hvidovre, Denmark). Five hundred nanograms RNA was reverse transcribed using the commercially available high-capacity cDNA reverse transcription kit [Applied Biosystems (AB), Foster City, CA, United States] to obtain cDNA for gene expression analysis.

### Quantitative Real-Time RT-PCR

Real-time RT-PCR was performed using custom TaqMan Low-Density Arrays (AB). cDNA equivalent to 125 ng total RNA mixed with Gene Expression Mastermix (AB) was loaded on to the arrays and run for 50 cycles in duplicates on the 7900 Sequence Detection System (AB). Data were collected and analyzed using SDS 2.4 software (AB). Technical duplicates were evaluated, and analyses were excluded when DCt > 1. Reference genes were verified using GeNorm software (Vandesompele et al., 2002). Data were expressed relative to the reference genes GAPDH and RPLP0 using the qBase^+^ software (Biogazelle, Zwijnaarde, Belgium). The following primers were used: VEGF-A: Hs00900054_m1; VEGF receptor 2 (VEGF-R2): Hs00911700_m1; hypoxia-inducible factor-1α (HIF-1α): Hs00936376_m1; heme oxygenase (HMOX)-1: Hs01110250_m1; matrix metalloproteinase (MMP)-2: Hs00957562_m1; MMP-9: Hs00957562_m1; glyceraldehyde-3-phosphate dehydrogenase: Hs99999905_m1; and 60S acidic ribosomal protein P0: Hs99999902_m1.

### Statistical Analysis

All datasets were analyzed using a linear mixed model, in which participant ID was defined as a random effect and time (baseline, mid, and post-intervention time points) and condition (BFRE, Control) as fixed effects. This model was used to evaluate within-group and between-group differences (i.e., baseline and time × group interactions). For datasets involving a single group (evaluation of within-group changes), participant ID was defined as a random effect and time as a fixed effect. All datasets were evaluated for model assumptions. Gene expression data were logarithmically transformed to conform with model assumptions. Data missing at random were imputed by the linear mixed model using maximum likelihood estimation. Data are presented as mean ± SD, except for gene expression data which are presented as geometric means ± SEM (back log transformed). Comparisons were considered statistically significant for *P* < 0.05 (two-tailed). All statistical analyses were performed using STATA 16.1 (StataCorp, College Station, TX, United States).

## Results

### Participants

Two participants from the BFR and one from the CON group left the project prematurely for reasons not related to the intervention procedures, resulting in 10 BFR and eight CON participants. Training data and exercise adherence have been reported elsewhere (Nielsen et al., 2012).

### Capillary Assessment

Capillaries per fiber (CF) increased with BFRE training from baseline (2.18 ± 0.64) to Mid8 (2.63 ± 1.00), Post3 (2.63 ± 0.49),and Post10 (2.58 ± 0.76) (*P* ≤ 0.05) (Figures 1, 2). These changes corresponded to relative increases of 24% (Mid8), 24% (Post3), and 21% (Post10), respectively. No changes were observed in CON during the intervention period (Figure 2).

Capillary density remained unchanged in both groups (Figures 1, 2). For mean CA, a between-group difference was observed at baseline (BFRE: 52.0 ± 7.1 μm^2^/CON: 64.7 ± 11.6) (*P* < 0.05). Furthermore, CA increased from baseline to Mid8 (67.6 ± 14.5 μm^2^), Post3 (69.6 ± 8.7 μm^2^), and Post10 (64.9 ± 11.6 μm^2^) (*P* ≤ 0.01) in response to BFRE training while remaining unchanged in CON (Figures 1, 2).

The ratio of total CA to myofiber area (CAMR) increased 15–17% from baseline (0.017 ± 0.003) to Mid8 (0.020 ± 0.003), Post3 (0.020 ± 0.002), and Post10 (0.020 ± 0.005) (*P* < 0.05), while an increase was also observed from baseline (0.020 ± 0.003) to Post3 (0.024 ± 0.004) for CON (Figure 2).

### Capillary and Pericyte Co-localization

A large degree of co-localization between CD31^+^ and NG2^+^ structures (≥99%) was observed at all time points with no apparent changes over time (Figure 1). When assessed as CD31^+^/NG2^+^-positive cells (DAPI^+^), a less abundant degree of co-localization was observed (39–44%), again with no changes over time in either group, except for a decrease in CD31^+^/NG2^+^-positive cells (DAPI^+^) per mm^2^ in CON at Post3 (*P* < 0.05). Due to the large degree of CD31 and NG2 co-localization, very few CD31^+^/NG2^–^ and CD31^–^/NG2^+^ cells (DAPI^+^) were observed (≤5%).

### Protein Expression

**FIGURE 3:**
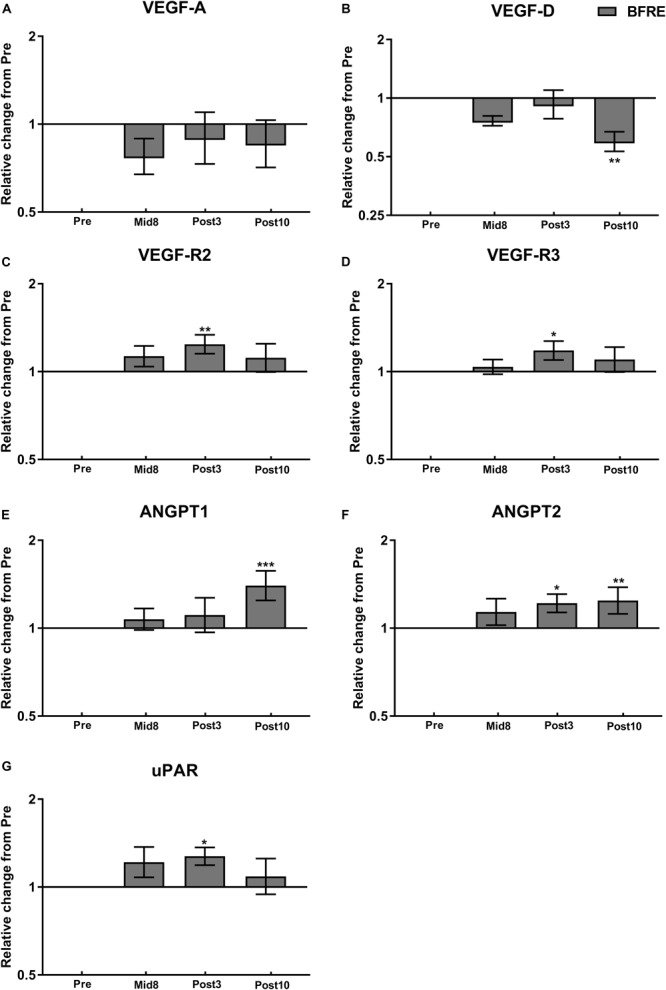
Protein expression related to angiogenesis signaling at baseline (Pre), 8 days into the training intervention (Mid8), and 3 and 10 days after cessation of training (Post3 and Post10, respectively). Dark gray bars denotes blood flow-restricted group. **(A)** Vascular endothelial growth factor A (VEGF-A), **(B)** VEGF-D, **(C)** VEGF receptor 2 (VEGF-R2), **(D)** VEGF-R3, **(E)** angiopoietin-1 (ANGPT1), **(F)** ANGPT2, and **(G)** urokinase-type plasminogen activator receptor (uPAR). Pre to Mid/post differences: ^∗^*P <* 0.05, ^∗∗^*P <* 0.01, ^∗∗∗^*P <* 0.001. Values are means ± SD; BFR: *n* = 10 at Mid8 *n* = 9.

VEGF-A protein remained unchanged from baseline in response to BFRE training, whereas VEGF-D protein decreased at Post10 (*P* < 0.05) (Figure 3). VEGF receptor 2 and 3 protein (VEGF-R2/3) increased Post3 (*P* < 0.05–0.01), which was also the case for urokinase-type plasminogen activator (uPA) receptor (uPAR) (*P* < 0.05) (Figure 3).

**FIGURE 4:**
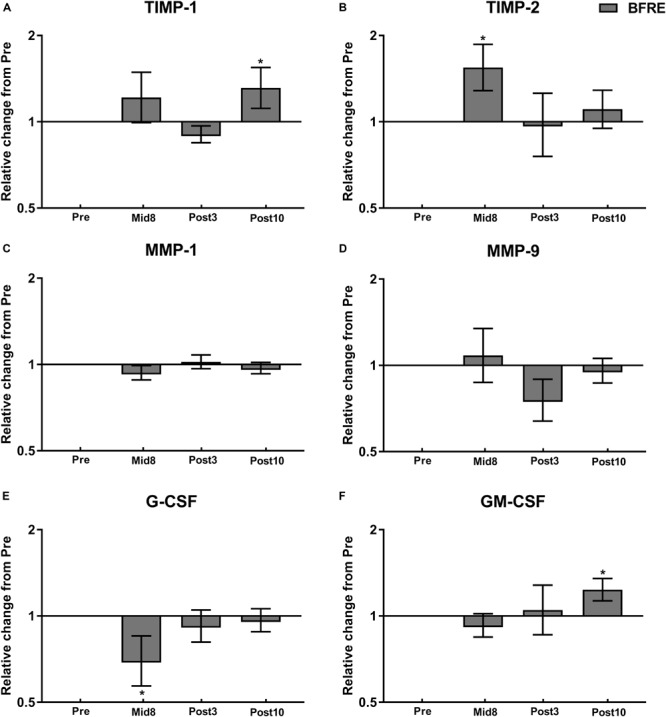
Protein expression related to vascular/extracellular matrix remodeling at baseline (Pre), 8 days into the training intervention (Mid8), and 3 and 10 days after cessation of training (Post3 and Post10, respectively). Dark gray bars denotes blood flow-restricted group. **(A)** Tissue inhibitor of matrix metalloproteinase (TIMP)-1, **(B)** TIMP-2, **(C)** matrix metalloproteinase (MMP)-1, **(D)** MMP-9, **(E)** granulocyte colony-stimulating factor (G-CSF), and **(F)** granulocyte-macrophage colony-stimulating factor (GM-CSF). Pre to Mid/post differences: ^∗^*P <* 0.05. Values are means ± SD; BFR: *n* = 10 at Mid8 *n* = 9.

Angiopoietin-1 (Ang-1) showed an increase Post10 (*P* < 0.05), while Ang-2 at Post3 and Post10 (*P* < 0.05–0.01) (Figure 4). Tissue inhibitor of matrix metalloproteinase (TIMP)-1 and TIMP-2 were upregulated Post10 and Mid8 (*P* < 0.05), respectively, whereas MMP-1 and MMP-9 remained unchanged during the time course of the intervention (Figure 4). Granulocyte colony-stimulating factor (G-CSF) decreased with BFRE training at Mid8 (*P* < 0.05), while granulocyte-macrophage colony-stimulating factor (GM-CSF) increased Post10 (*P* < 0.05) (Figure 4).

### mRNA Expression

**FIGURE 5:**
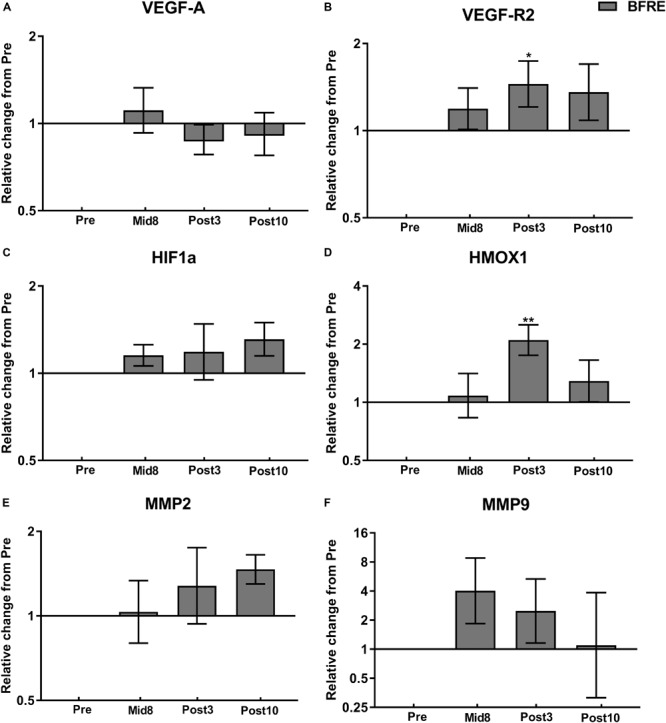
mRNA expression related to angiogenesis and vascular/matrix remodeling at baseline (Pre), 8 days into the training intervention (Mid8), and 3 and 10 days after cessation of training (Post3 and Post10, respectively). Dark gray bars denotes blood flow-restricted group. **(A)** Vascular endothelial growth factor A (VEGF-A), **(B)** hypoxia-inducible factor-1α (HIF-1α), **(C)** VEGF receptor 2 (VEGF-R2), **(D)** heme oxygenase (HMOX)-1, **(E)** matrix metalloproteinase (MMP)-2, and **(F)** MMP-9. Pre to Mid/post differences: ^∗^*P <* 0.05, ^∗∗^*P <* 0.01. Values are geometric means ± SEM; BFR: *n* = 10 at Mid8 *n* = 9.

VEGF-A and HIF-1α mRNA remained unchanged at all time points, whereas an increase in VEGF-R2 and HMOX-1 mRNA was observed following BFRE training at Post3 (*P* < 0.05) (Figure 5). MMP-2 and MMP-9 remained unchanged with BFRE training (Figure 5).

### Perivascular Basal Membrane Analysis

Following BFRE training, a single participant demonstrated a small increase in focal pBLi at Mid8 relative to baseline. More marked changes in pBLi were noted at Post3, where small/moderate/large gains in immunoreactivity relative to baseline were observed in 4/1/1 participants, respectively, while 3/2 participants showed small/moderately elevated pBLi levels at Post10 (see Figure 1 for representative images). A single participant in CON showed slightly increased pBLi relative to baseline at Mid8, with no other changes observed following free-flow work-matched training (CON).

## Discussion

This study is the first to report peripheral microvascular adaptations in response to low-load BFRE resistance training in humans. The present data demonstrate a consistent increase in capillary number per myofiber (C:F) and CA with BFRE training, altogether resulting in an increased overall CA per mm^2^ muscle (elevated CAMR). Thus, high-frequency low-load BFRE training appears to effectively stimulate angiogenesis and increase skeletal muscle perfusion capacity in young healthy individuals.

### Microvascular Properties

Notably, an increase in the C:F ratio was observed 3 and 10 days following cessation of BFRE training, indicating remodeling of microvascular muscle properties. The observed increase in C:F ratio was paralleled by a proportional increase in myofiber area (Nielsen et al., 2012) resulting in a stable CD throughout the study period, suggesting that the increase in capillary content may primarily serve to support the training-induced increase in myofiber area. A 25–34% increase in CA was also observed with BFRE training, likely a result of an increase in capillary lumen and/or endothelial cell swelling (discussed in detail below). The increases in capillary lumen and C:F ratio altogether resulted in a 15–17% increased CMAR, which suggests an improved skeletal muscle perfusion capacity. In support of CA as a valid proxy of the capillary lumen, previous studies have shown capillary lumen to increase without changes in endothelial cell area in hypertensive patients (Gliemann et al., 2015) and in mice (Baum et al., 2018) following 8 weeks and 1 week of endurance training, respectively. In the present study, a consistent increase in CMAR was noted in response to BFRE training at all time points as well as with free-flow training (CON) at Post3. Increases in C:F and CA would be expected to result in improvements in peripheral diffusion area (i.e., CMAR), which theoretically would ensure improved vascular conductance and prolonged mean transit time during exercise (provided that capillary lengths and muscle blood flow remain unchanged) in turn potentially facilitating O_2_ extraction, nutrient exchange, and heat removal from the active myofibers. On the other hand, capillary length may also change as a result of physiological adaptations to exercise; i.e., improvements in capillary tortuosity have previously been reported in response to endurance training both in human and mice skeletal muscle (Charifi et al., 2004; Baum et al., 2018). Unfortunately, we were not able to analyze capillary tortuosity in the present study; however, even if capillary lengths were to increase with BFRE training in the present study, this would be unlikely to cancel the positive effect of an increased capillary lumen, as the capillary radius is weighted substantially stronger (radius^4^) than capillary length (length^1^) in that relationship. Thus, it may be speculated that the present increase in CMAR reflects an enhanced perfusion capacity per skeletal muscle area, which potentially could contribute to the improved endurance capacity previously reported with BFR training (Abe et al., 2010; Kacin and Strazar, 2011).

Alternatively, the increase in CA could also reflect endothelial cell swelling, as occasionally reported in response to strenuous exercise training (Brzank and Pieper, 1986; Hudlicka et al., 2003) and also observed with acute protocols of prolonged ischemia (30–180 min) and subsequent reperfusion (Gidlof et al., 1988; Ward and Mccarthy, 1995; Hudlicka et al., 2008). Endothelial cell swelling might even cause narrowing in the capillary lumen and in turn reduce peripheral flow (Kretschmar and Engelhardt, 1994; Mazzoni et al., 1995). The mechanism(s) involved in endothelial cell swelling is thought to involve formation of reactive oxygen species (ROS), as endothelial thickening induced by chronic ischemia appears to be attenuated by endogenous inhibition of ROS (Hudlicka et al., 2008).

Collectively, the consistent reports of absent or limited ROS formation (Nielsen et al., 2017a; Centner et al., 2018; Patterson et al., 2019), indirect signs of elevated blood flow (Evans et al., 2010; Hunt et al., 2013), and improved endurance capacity (Abe et al., 2010; Kacin and Strazar, 2011) in response to low-load BFRE/training suggest that the observed increase in CA primarily arises from increased capillary lumen rather than endothelial cell thickening/hypertrophy. However, more detailed analysis (e.g., electron microscopy) is warranted to provide solid conclusions on these aspects.

The present changes in peripheral microvascular properties in response to short-term (3 weeks, twice daily) BFRE training are striking, as comparable adaptations (i.e., 15–17% gains in C:F) normally require 2–3 months of endurance or strength training intervention to take place (Klausen et al., 1981; Green et al., 1999). A lack of change in C:F and CD in response to 6 weeks of low-frequency (three sessions/week) bodyweight squat BFR training was recently reported by Jakobsgaard et al. (2018). In contrast, training-induced increases in C:F ratio were observed in type I myofibers in response to alternating blocks (weeks) of high-frequency (daily) BFR training in weightlifters (Bjornsen et al., 2019). The contradicting results may at least partly be related to the study differences in exercise frequency, cuff pressure, and duration, respectively. Notably, vascular adaptations were observed to occur with moderate occlusion pressure (100–120 mmHg), high-frequency (one to two sessions a day), and 2–3 weeks of BFRE (present data, Bjornsen et al., 2019) in contrast to using low-frequency (three sessions/week) BFRE at high cuff pressures (100 rising to 180 mmHg) for 6 weeks’ duration. Notably, the present data suggest that microvascular adaptations emerged already in the early intervention phase (Mid8). Further, the present pBLi data along with previous observations of elevated calcium/calmodulin-dependent protein kinase (CaMK)II expression (Nielsen et al., 2017b) as well as increased macrophage content and upregulated heat shock protein (HSP)27 expression (Nielsen et al., 2017a) indicate that when performed for longer duration (>1–2 weeks), strenuous BFRE training (i.e., performed to task failure, high cuff pressure) may lead to vascular and myocellular stress.

Previously, increases in C:F ratio have been a general observation following after conventional heavy-load resistance training (Mccall et al., 1996; Holloway et al., 2018). Similar to the present data, these studies observed equivalent increases in myofiber fiber area and C:F ratio, altogether resulting in unchanged CD (Mccall et al., 1996; Green et al., 1999; Holloway et al., 2018). Notably, Holloway et al. (2018) reported C:F to increase after just 2 weeks of high-volume resistance training, which together with the present data underline the early biological efficacy of exercise-induced angiogenesis. Likewise, early signs of improved capillarization (i.e., increases in C:F and CD) were observed in response to 4 weeks of moderate- to high-intensity endurance training (Jensen et al., 2004; Hoier et al., 2012), overall supporting that a high degree of microvascular plasticity may exist in the very initial phase of exercise training.

### Molecular Signaling for Angiogenesis

VEGF-A is considered a key stimulator of angiogenesis in skeletal muscle, while HIF-1α is known to act as an important regulator of VEGF-A expression (Hoier and Hellsten, 2014). Somewhat surprisingly, therefore, VEGF-A and HIF-1α protein and mRNA levels remained unchanged in the present study despite significant alterations in capillarization. Increases in VEGF-A and HIF-1α mRNA expression have previously been observed in response to acute bouts of BFRE (Larkin et al., 2012; Ferguson et al., 2018), whereas less consistent data exist on VEGF-A protein abundance as manifested by no changes in muscle and plasma (Larkin et al., 2012) or increases in plasma after acute BFRE (Takano et al., 2005; Patterson et al., 2013) and no change in muscle in response to 6-week BFRE (Jakobsgaard et al., 2018). Notably, VEGF-A and HIF-1α expressions seem to be upregulated acutely at the mRNA level in response to exercise (Hiscock et al., 2003; Hoier et al., 2012) and typically return to baseline levels ∼20 h post-exercise (Hiscock et al., 2003; Larkin et al., 2012), whereas VEGF-A protein content measured in muscle biopsies often remain unchanged in response to acute exercise (Hoier et al., 2012; Hoier and Hellsten, 2014) potentially due to high levels of secretion to the interstitium and circulation (Hiscock et al., 2003; Hoier et al., 2012; Hoier and Hellsten, 2014). Thus, the relatively late time points of muscle biopsy sampling (≥3 days post-exercise) in the present study may have prevented detecting any acute elevations in VEGF-A signaling. Furthermore, given the pronounced increase in microvascular adaptation at Mid8, we may have missed to detect increases in angiogenic signaling in the very initial intervention (pre-to-Mid8). Regardless, the present upregulation in VEGF-R2 and -R3 protein levels and VEGF-R2 mRNA at Post3 suggests that some sensitization of the VEGF-related angiogenesis signaling pathway may indeed have occurred with BFRE training. Further, the increase in HMOX-1 mRNA observed at Post3 suggests that VEGF signaling may have been elevated during the time course of BFRE training. VEGF-A is known to stimulate HMOX-1 expression and activity in endothelial cells, after which HMOX-1 can induce endothelial cell VEGF-A synthesis and increase VEGF-A activity, resulting in a positive feedback loop (Jozkowicz et al., 2002). In addition, it can be speculated that the BFR-induced increase in myofiber area (Nielsen et al., 2012) and the present signs of capillary neoformation are casually interrelated, as the overexpression of AKT-related hypertrophy signaling in rodent (mice) skeletal muscle has been shown to increase the synthesis of HMOX-1 in endothelial cells and resident macrophages, respectively, in turn facilitating angiogenesis (Onoue et al., 2018).

### Signaling for ECM Remodeling

In addition to angiogenesis signaling, remodeling of the capillary network and ECM is essential for microvascular plasticity (Gustafsson, 2011). The MMPs and angiopoietins play an important role in the regulation of vascular remodeling (Gustafsson, 2011; Haas and Nwadozi, 2015). The present observation of no changes in MMP protein/mRNA with BFRE training (i.e., non-significant two/fourfold changes in MMP-2/9 mRNA) suggests that no major changes in ECM composition and/or structure took place, which is further supported by the two-fold increase in protein abundance of the MMP inhibitor, TIMP-2, at Mid8.

On the other hand, the present increase in Ang-2 at Post3 and Post10 and uPAR protein content at Post3 are indicative of some level of vascular remodeling. An increase in Ang-2 in parallel with available VEGF-A stimulates the angiogenic process through Ang-2-induced endothelial destabilization, whereas elevations of Ang-2 in absence of VEGF-A can lead to capillary degradation (Gustafsson, 2011; Fagiani and Christofori, 2013). Furthermore, Ang-2 also appears to stimulate VEGF-A production in endothelial/myocyte cell cultures (Gorman et al., 2014). The present observation of increased uPAR content, the receptor of the uPA, following 3 weeks of BFRE training indicates that this training modality may activate the uPA system.

Notably, the uPA system has been shown to facilitate angiogenesis through pericellular proteolysis resulting in increased vascular permeability of the endothelial membrane, in turn allowing for the proliferation and migration of endothelial cells (Breuss and Uhrin, 2012). Finally, vascular neogenesis and ECM remodeling may also have been facilitated by elevations in M2-macrophage content (Jetten et al., 2014), which we observed predominantly in close proximity to the vascular compartment following the period of BFRE training (Nielsen et al., 2017a).

### Perivascular Membrane Properties

Notably, clear indications of increases in pBLi were observed at all time points in response to BFRE training, while largely no changes emerged with free-flow work/load-matched training (CON). To our best knowledge, training-induced increases in pBLi have not been reported in the literature previously, but it may reflect a thickening of the perivascular basal membrane. Notably, perivascular basal membrane thickening has been observed using electron microscopy in various pathophysiological conditions including diabetes mellitus and in chronically hypertensive patients (Raskin et al., 1983; Sosenko et al., 1984; Peschen et al., 1996; Gliemann et al., 2015), whereas basal membrane thinning seems to be an adaptation to endurance training in healthy older individuals as well as diabetic and hypertensive patients (Williamson et al., 1996; Gliemann et al., 2015). Perivascular basal membrane thickening has been speculated to be stimulated by severe local hypoxia and/or elevated blood pressure as well as chronic inflammation (Baum and Bigler, 2016), the former factor suggesting a potential link to BFRE. Notably, focal signs of basal membrane thickening were mainly observed at Post3 in participants engaging in BFRE training, suggesting that this condition was stimulated by strenuous BFRE training (one to two daily exercise sessions performed to task failure for 3 weeks). However, only a few participants demonstrated pronounced basal lamina thickening (moderate/high), and importantly this effect appeared transient as these cases overall showed bettering (reduced basal lamina thickening) at Post10. Yet, several BFRE participants (five of 10) showed some sign of basal lamina thickening 10 days after the cessation of the intervention, which may be of some concern as the functional consequence may be restricted blood/tissue diffusion, reduced leukocyte blood-to-tissue migration, and impaired shear stress-induced angiogenesis (Baum and Bigler, 2016).

### Methodological Considerations

Timing of the post-training muscle biopsy sampling (3 days post-exercise) might have limited the information gained from protein signaling and mRNA analysis, as angiogenesis signaling (protein/mRNA level) generally appears transiently upregulated within a 24-h window post-exercise. In addition, the lack of protein signaling and mRNA data from controls performing work-matched free-flow training limits the interpretation of these data.

Furthermore, the concurrent increases observed in C:F and CA could be casually interrelated, as an increase in CA would theoretically lead to an elevated capillary (C:F) detection rate in any given biopsy cross section.

## Conclusion

In conclusion, the present study presents novel evidence that short-term high-frequency BFRE training leads to angiogenesis as manifested by elevated capillary number and cap-to-muscle area ratio, potentially leading to improvements in microvascular perfusion capacity. As such, these observations provide important information about the effect and adaptive mechanisms of hypoxic exercise-induced angiogenesis.

Indications of increases in capillary basement membrane thickness were noted, which potentially could lead to reductions in myocellular oxygen diffusion capacity, heat exchange, and nutrient delivery. These observations warrant further awareness in future experimental settings.

## Data Availability Statement

The datasets generated for this study are available on request to the corresponding author.

## Ethics Statement

The studies involving human participants were reviewed and approved by the Research Ethical Committee of Southern Denmark. The patients/participants provided their written informed consent to participate in this study.

## Author Contributions

JN, PA, and UF contributed to the conception and design of the study. JN, PA, KJ, TP, LD, RB, TN, CS, and UF contributed to the collection, analysis, and interpretation of data and drafting the article or revising it critically for important intellectual content. All authors were involved in revisions and accepted the final, submitted version of the manuscript. All persons designated as authors qualify for authorship, and all those who qualify for authorship are listed. All data collection and analysis were carried out at the Department of Sports Science and Clinical Biomechanics, University of Southern Denmark, Odense, Denmark.

## Conflict of Interest

The authors declare that the research was conducted in the absence of any commercial or financial relationships that could be construed as a potential conflict of interest.

## Abbreviations

BFR: blood flow restriction.
